# A Growing Nuisance

**Published:** 1877-11

**Authors:** 


A Growing Nuisance.—The please-send-me-a-specimen-copy-of-your-valuable-journal-man is plainly on the increase. He is becoming more frequent; in fact, he is without number and ubiquitous.

In former days he hid his request—but never a stamp for return postage—in an envelop. Latterly he selects the curtly business "postal" card, thereby cheapening the cost to him a couple of coppers, and making an inclosure impossible. In all the years of our editorial life—and we trust our "sands have" not yet "run out" by a long shot—we have never seen the color of the send-me-a-specimen-copy-man's money. We are beginning to fear we never shall. Hope, indeed, has been so long deferred, that it has fled our breasts entirely.

Musing, the other day, this please-send-me, etc., pest—this unknown but oft-recurring nuisance—rose to our mind's eye as he cut the wrappers from the American Practitioner, the New York Medical Journal, and the St. Louis Medical Journal, all reaching him by the same mail. For "no pent-up Utica" puts a limit to the demands of this profitless patron. He longs to hear from the several sections of our broad realm. His vocabulary contains no north, no south, etc., but embraces the boundless continent. His love of knowledge is so intense that it swallows up every other consideration, thought, principle. In another month he will draw on the sunny south—very sunny just now—for his supplies. And then he is as impartial as he is insatiable. His cravings will demand in turn the Nashville, the New Orleans, and the Atlanta journals. An occasional quarterly is needed to sandwich any failure of the mails; and the swift-recurring weekly is, alas! doubtless taken in vast numbers as a "condignment," as poor Artemus Ward used to say of mustard. But with all his gettings, this chronic abomination has never gotten his dues, which would be exposure. Who will see that he has them? We will. No more postals, O man without bowels! but enclose the fractional quarter, and then get your reading without filching from your betters.—*American Practitioner.*

